# Therapeutic Effects of the Superoxide Dismutase Mimetic Compound Mn^II^Me_2_DO2A on Experimental Articular Pain in Rats

**DOI:** 10.1155/2013/905360

**Published:** 2013-09-01

**Authors:** Lorenzo Di Cesare Mannelli, Daniele Bani, Andrea Bencini, Maria Luisa Brandi, Laura Calosi, Miriam Cantore, Anna Maria Carossino, Carla Ghelardini, Barbara Valtancoli, Paola Failli

**Affiliations:** ^1^Department of Pharmacology, University of Florence, Viale Pieraccini 6, 50139 Florence, Italy; ^2^Department of Anatomy, Histology and Forensic Medicine, University of Florence, Viale Pieraccini 6, 50139 Florence, Italy; ^3^Department of Chemistry, University of Florence, Via della Lastruccia 3, 50019 Sesto Fiorentino, Florence, Italy; ^4^Department of Internal Medicine, University of Florence, Viale Pieraccini 6, 50139 Florence, Italy

## Abstract

Superoxide anion (O_2_^•−^) is overproduced in joint inflammation, rheumatoid arthritis, and osteoarthritis. Increased O_2_^•−^ production leads to tissue damage, articular degeneration, and pain. In these conditions, the physiological defense against O_2_^•−^, superoxide dismutases (SOD) are decreased. The Mn^II^ complex MnL4 is a potent SOD mimetic, and in this study it was tested in inflammatory and osteoarticular rat pain models. *In vivo* protocols were approved by the animal Ethical Committee of the University of Florence. Pain was measured by paw pressure and hind limb weight bearing alterations tests. MnL4 (15 mg kg^−1^) acutely administered, significantly reduced pain induced by carrageenan, complete Freund's adjuvant (CFA), and sodium monoiodoacetate (MIA). In CFA and MIA protocols, it ameliorated the alteration of postural equilibrium. When administered by osmotic pump in the MIA osteoarthritis, MnL4 reduced pain, articular derangement, plasma TNF alpha levels, and protein carbonylation. The scaffold ring was ineffective. MnL4 (10^−7^ M) prevented the lipid peroxidation of isolated human chondrocytes when O_2_^•−^ was produced by RAW 264.7. MnL4 behaves as a potent pain reliever in acute inflammatory and chronic articular pain, being its efficacy related to antioxidant property. Therefore MnL4 appears as a novel protective compound potentially suitable for the treatment of joint diseases.

## 1. Introduction

Reactive oxygen species (ROS) are by-products of cellular metabolism and can behave as second messengers in physiological conditions. However, in degenerative and inflammatory diseases, ROS production is dramatically increased and can induce cell, tissue, and organ toxicity [1]. In particular, ROS overproduction is a typical hallmark of rheumatoid arthritis [2, 3] and osteoarthritis [4, 5]. In addition, ROS are involved in pain sensation [6–9].

Superoxide anion (O_2_^•−^) is one of the most harmful oxidant species identified in the above pathological conditions [2]. The superoxide dismutase enzymes (SOD) can reduce O_2_^•−^ toxicity. Three SOD families have been characterized: the cytosolic Cu/Zn-SOD1, the matrix mitochondrial Mn-SOD2, and the extracellular EC-SOD3.

The importance of ROS in joint degeneration is indicated by the finding that EC-SOD3-deficient mice show increased severity of collagen-induced arthritis [10]. Moreover, in inflammatory conditions, O_2_^•−^ reacts with nitric oxide to form peroxynitrite which can decrease SOD functionality [9]. In addition, Mn-SOD2 is downregulated in osteoarticular cartilage [11, 12]. EC-SOD3 is also decreased in the cartilage of osteoarthritic patients and in a mouse model of osteoarthritis [13]. Of note, decreased SOD2 and SOD3 expression precedes the appearance of histological lesions in osteoarticular cartilage [14]. 

All these data emphasize the concept that, during inflammation and degenerative arthritis, the physiological defences against O_2_^•−^ are reduced, suggesting that compounds able to decompose O_2_^•−^ may be pharmacological aids for the treatment of articular pain.

We have described the O_2_^•−^ scavenging activity of some polyamine-polycarboxylate-Mn^II^ complexes [15]. Among tested compounds, the 4,10-dimethyl-1,4,7,10 tetraazacyclododecane-1,7-diacetic acid Mn^II^ complex (Mn^II^Me_2_DO2A, herein indicated as MnL4, Supplementary Material, See Figure S1 in Supplementary Material available online at http://dx.doi.org/10.1155/2013/905360) is the most potent agent of the series.

In a cellular environment, MnL4 (1 *μ*M–10 nM) dose-dependently reduces O_2_^•−^ generated enzymatically (xanthine/xanthine oxidase) or by formyl-methionyl-leucyl-phenylalanine- (fMLP-) activated macrophages. MnL4 (100 nM) can cross cell membranes and significantly reduces oxidative injury in cells exposed to O_2_^•−^. Systemically administered to mice (5–15 mg kg^−1^ body weight), MnL4 reduces the acute pain induced by acetic acid (writhing test). Since this anti-inflammatory effect has been observed with both intraperitoneal and oral administration, MnL4 demonstrates a favourable pharmacokinetic profile [15]. Moreover, MnL4 markedly reduces lung inflammation, oxidative, injury, and breathing dysfunction induced by exposure to the airborne allergen in sensitized guinea pigs [16].

Compared with other SOD mimetics, MnL4 would have the advantage of being a smaller, more lipophilic molecule, capable of readily entering cells and decomposing O_2_^•−^ at cytoplasmic sites of generation [17]. The present study was therefore designed to test the action of MnL4 in rodent models currently used to reproduce acute inflammation, rheumatoid arthritis, or osteoarthritis. According to the high potency of this SOD mimetic compound, we used the low dosage 15 mg kg^−1^.

## 2. Materials and Methods

### 2.1. Animals

Male Sprague-Dawley rats (Harlan, Varese, Italy) weighing approximately 200–250 g at the beginning of the experimental procedure were used for the experiments. Four animals per cage were housed at 23 ± 1°C under a 12 h light/dark cycle; they were fed with standard laboratory diet and tap water *ad libitum* and used at least one week after their arrival. The experimental protocol complied with the European Community guidelines for animal care (DL 116/92, the European Communities Council Directive of 24 November 1986: 86/609/EEC) and was approved by the animal subject reviews' board of the University of Florence. The ethical policy of the University of Florence complies with the Guide for the Care and Use of Laboratory Animals of the US National Institutes of Health (NIH Publication no. 85-23, revised 1996; University of Florence assurance number: A5278-01). Animals were anesthetized with 2% isoflurane before the surgical procedures and sacrifice, which was performed by cervical dislocation. All efforts were made to minimize suffering and reduce the number of animals used. Rats were randomly assigned to each experimental group and individually habituated to handling before testing.

### 2.2. Drug Administration

MnL4, H_2_L4, diclofenac, ibuprofen, and gabapentin were dissolved in sterile saline solution. In a first experimental set, the abovementioned compounds were administered acutely by intraperitoneal (i.p.) injections at the indicated doses. MnL4 and H_2_L4 dosages were chosen on the base of previous experiments [15]; typical doses were chosen for diclofenac, ibuprofen, and gabapentin [18–20].

In a second experimental set, MnL4 was given by continuous subcutaneous (s.c.) delivery using an osmotic minipump (Alzet 2002, Palo Alto, CA, USA) implanted on the back and filled to deliver a daily dose of 15 mg kg^−1^ for 14 days. 

The organic ligand 4,10-dimethyl-1,4,7,10-tetraazacyclododecane-1,7-diacetic acid (H_2_L4) was synthesized as previously reported. This compound was isolated as trihydrochloride salt (H_2_L4·3HCl [15]). Its Mn^II^ complex, MnL4, was obtained by reaction of MnSO_4_ with H_2_L4·3HCl (1 : 1 molar ratio) in aqueous solution at neutral pH under nitrogen atmosphere; the MnL4 complex was then isolated as a white solid by precipitation with an ethanol/diethyl ether 2 : 1 mixture, according to a previously described procedure [15]. The complex was further purified by recrystallization with water/ethanol. The purity of both compounds was ascertained by elemental analysis. Diclofenac, ibuprofen, gabapentin, and fMLP were purchased from Sigma-Aldrich, Milan, Italy.

### 2.3. Carrageenan-Induced Acute Inflammatory Pain

100 *μ*L of carrageenan solution (Sigma-Aldrich; 1% in saline) was injected intraplantarly into the left hindpaw. Three hours after carrageen injection, MnL4, H_2_L4 (15 mg kg^−1^), diclofenac, ibuprofen (15 and 100* *mg kg^−1^), or saline was i.p. administered and their antihyperalgesic effect was measured along the following 45 minutes (at time 15, 30, and 45 minutes) by the paw pressure test. Control rats received 100 *μ*L of saline solution intraplantarly and saline i.p.

### 2.4. Freund's Adjuvant-Induced Inflammatory Arthritis

Articular damage was induced by injection of complete Freund's adjuvant (CFA, Sigma-Aldrich) into the tibiotarsal joint [21]. Briefly, the rats were slightly anesthetized by 2% isoflurane, the left leg skin was sterilized with 75% ethyl alcohol, and the lateral malleolus located by palpation; then, a 28-gauge needle was inserted vertically to penetrate the skin and turned distally for insertion into the articular cavity at the gap between the tibiofibular and tarsal bone until a distinct loss of resistance was felt. A volume of 50 *μ*L of CFA was then injected (day 0). The paw pressure and the incapacitance tests (see below) were performed 7 days after CFA administration. MnL4 (5 and 15* *mg kg^−1^), H_2_Me_2_DO2A (15* *mg kg^−1^), ibuprofen (15 and 100 mg kg^−1^), diclofenac (15 and 100 mg kg^−1^), or saline was i.p. administered. Control rats received 50 *μ*L of saline solution (day 0) in the tibiotarsal joint and saline i.p. at day 7.

### 2.5. Monoiodoacetate-Induced Osteoarthritis

Unilateral osteoarthritis was also induced by injection of monoiodoacetate (MIA, Sigma-Aldrich) into the tibiotarsal joint. On day 0, rats were slightly anesthetized by 2% isoflurane, the left leg skin was sterilized with 75% ethyl alcohol, and the lateral malleolus located by palpation; then, a 28-gauge needle was inserted vertically to penetrate the skin and turned distally for insertion into the articular cavity at the gap between the tibiofibular and tarsal bone until a distinct loss of resistance was felt. 2 mg MIA in 25 *μ*L saline was delivered into the left articular cavity. The paw pressure and the incapacitance tests (see below) were performed at day 14. MnL4 (15* *mg kg^−1^), H_2_Me_2_DO2A (15* *mg kg^−1^), gabapentin (70* *mg kg^−1^), or saline was i.p. administered. Control rats received 25 *μ*L of saline solution (day 0) in the tibiotarsal joint and saline i.p. at day 14. To evaluate its preventive effect, MnL4 was administered by continuous s.c. infusion, from day 0 to day 14, using the Alzet 2002 osmotic minipump (15* *mg kg^−1^ day). 

### 2.6. Paw Pressure Test

The pain threshold in the rat was determined with an analgesimeter (Ugo Basile, Varese, Italy) as described [22]. Briefly, a constantly increasing pressure was applied to a small area of the dorsal surface of the paw using a blunt conical probe. Pressure was increased until a vocalization or a withdrawal reflex occurred. The withdrawal threshold was expressed in grams, the test was repeated twice, and the mean was considered as the value for each paw. Before starting experimental protocols, pain threshold was evaluated and rats scoring below 50 g or over 80 g were discarded. These limits assured a more precise determination of mechanical withdrawal threshold in experiments aimed to determine the effect of treatments. Mechanical pressure application was stopped at 150 g independently of rat reflex. Blind experiments were performed. In the saline + saline, carrageenan + saline, CFA + saline, and MIA + saline treated groups, recorded pressure values did not vary when repetitively measured during the experimental session. 

### 2.7. Incapacitance Test

Weight bearing changes were measured using an incapacitance apparatus (Linton Instrumentation, UK) detecting changes in postural equilibrium after a hind limb injury [23]. Rats were trained to stand on their hind paws in a box with an inclined plane (65° from horizontal). This box was placed above the incapacitance apparatus. This allowed us to independently measure the weight that the animal applied on each hind limb. The value considered for each animal was the mean of 5 consecutive measurements. In the absence of hind limb injury, rats applied an equal weight on both hind limbs, indicating a postural equilibrium, whereas an unequal distribution of the weight on hind limbs indicated a monolateral decreased pain threshold. Data are expressed as the difference between the weight applied on the limb contralateral to the injury and the weight applied on the ipsilateral one. Blind experiments were performed.

### 2.8. Histopathological and Biochemical Evaluations

Tissues of rats used to study the preventive effect of MnL4 (minipump infused) and their controls were analyzed as follows. (a) Legs were cut under the knee, flayed, and fixed in 4% formaldehyde in phosphate-buffered saline (PBS) for 48 h. Samples were then rinsed in PBS and placed in decalcifying solution (4N formic acid in distilled water), which was changed every 7 days until bone demineralization was complete (42 days). Samples were dehydrated in graded ethanol, embedded in paraffin wax, cut into 6 *μ*m thick sections, and stained with hematoxylin and eosin. Histological sections taken in the midst of the tibiotarsal joint were viewed and photographed under a light microscope equipped with a digital camera. (b) After sacrifice, blood was collected in heparin-treated tubes and plasma fraction was isolated by centrifugation. Plasmatic TNF-*α* levels were evaluated by ELISA method (eBioscience, San Diego, CA, USA), using a specific antirat polyclonal antibody. In order to obtain detectable levels of TNF plasma samples were lyophilized and reconstituted in 1/5 of the initial volume. Range sensitivity was 11.2–2.500 pg mL^−1^. (c) Total plasma proteins was quantified by bicinchoninic acid (BCA; Sigma-Aldrich) assay. Then, 20 *μ*g of each sample was denatured by 6% SDS and derivatized by 15 min incubation with 2–4 dinitrophenyl hydrazine (DNPH; Sigma-Aldrich) at room temperature in order to evaluate carbonylated protein evaluation. Samples were separated on a 10% sodium dodecyl sulphate- (SDS-) polyacrylamide gel by electrophoresis and blotted onto nitrocellulose membranes (BioRad, Milan, Italy). Membranes were blocked with 5% non-fat dry milk in phosphate-buffered saline (PBS) containing 0.1% Tween 20 (PBST) and then incubated overnight with anti-DNPH primary antibodies (Sigma-Aldrich; 1 : 5000 in PBST added with 5% non-fat dry milk). After washing with PBST, the membranes were incubated for 1 h in PBST containing the appropriate horseradish peroxidase-conjugated secondary antibody (1 : 5000; Cell Signalling, USA) and thoroughly washed [24]. The chemiluminescent substrate ECL (Pierce, USA) was used to visualize the peroxidase-coated bands. Densitometric analysis was performed using the free-share Scion Image 4.03 image analysis software (Scion Corp., Frederick, MD, USA). Ponceau-stained membranes were used as loading control [25].

### 2.9. Patient's Characteristics and Isolation of Human Chondrocytes

Human chondrocytes used for the experiments were isolated from 3 patients requiring arthroplasty for degenerative disorders of the knee. Slices of articular cartilage were obtained from a peripheral zone of the affected joint, outside regions with macroscopic degeneration but close to the calcified cartilage layer, after administration of an informed consent approved by the Local Ethical Committee.

Human chondrocytes (HCs) were isolated and cultured in Dulbecco's Modified Eagle's Medium (DMEM) supplemented with 10% fetal calf serum (FCS, Gibco, Invitrogen, Italy), 2 mM L-glutamine, 100 IU mL^−1^ penicillin, and 100 *μ*g mL^−1^ streptomycin in 5% CO_2_ atmosphere at 37°C as described [26]. 

### 2.10. Human Chondrocyte Lipid Peroxidation Induced by Stimulated RAW264.7

For the experiments, HCs at the 3rd culture passage were used in 5 separate experiments. They were grown to 90% confluence on 6-well cell culture plates (Corning, Italy) and starved for 18 h in serum-free medium. The mouse leukemic monocyte macrophage cell line (RAW 264.7) was obtained from American Type Culture Collection (Rockville, MD, USA). RAW 264.7 were grown in DMEM supplemented with 10% FCS, 2 mM L-glutamine, 100 IU mL^−1^ penicillin, and 100 *μ*g mL^−1^ streptomycin in 5% CO_2_ atmosphere at 37°C. 72 h before experiments, cells were detached, plated on the upper layer of polycarbonate transwell dishes with a pore diameter of 3 *μ*m (Corning, Italy), and starved in serum-free medium for the last 18 h. The transwells were then placed into HCs-containing wells, and cells were coincubated together in DMEM without phenol red for 30 min in the absence or presence of 10^−7^ M MnL4. Then, RAW 264.7 were activated with 10^−7^ M fMLP (dissolved in DMEM), while in control samples the same volume of DMEM was added. According to previous data [15], fMLP-activated RAW 264.7 produced a significant and reproducible amount of O_2_^•−^. The basal value of HCs lipid peroxidation was obtained in cells not cocultured with RAW 264.7. After 4 h, the reaction was stopped on ice; the upper layer with RAW 264.7 was removed. HCs were scraped in 1 mL of cold PBS, and the cell suspension was used to measure the thiobarbituric acid reactive substances (TBARS), assumed as a marker of cell oxidative injury. Briefly, the suspensions were mixed with 4 mL thiobarbituric acid (36 mM in acetic acid/sodium acetate, adjusted to pH 4 with NaOH) and boiled for 1 h. After cooling on ice, the mixture was centrifuged at 5000 ×g for 10 min and the absorbance of the supernatant was spectrophotometrically evaluated at the 532 nm wavelength against a standard curve of 1,1,3,3-tetramethoxypropane. Protein concentration in the samples was determined using the Coomassie protein assay (Pierce, Rockford, IL, USA). TBARS values were expressed as *μ*mol mg^−1^ of proteins. All reagents used were of the highest purity grade. 

### 2.11. Statistical Analysis

All experiments were evaluated blind. Results were expressed as the means ± s.e.m. Statistical analysis of differences among the experimental groups was performed using one-way ANOVA followed by Student-Newman-Keuls *post hoc* test. A P value ≤ 0.05 was considered significant.

## 3. Results

### 3.1. Effects of MnL4 on Carrageenan-Induced Acute Inflammatory Pain

**Figure 1:**
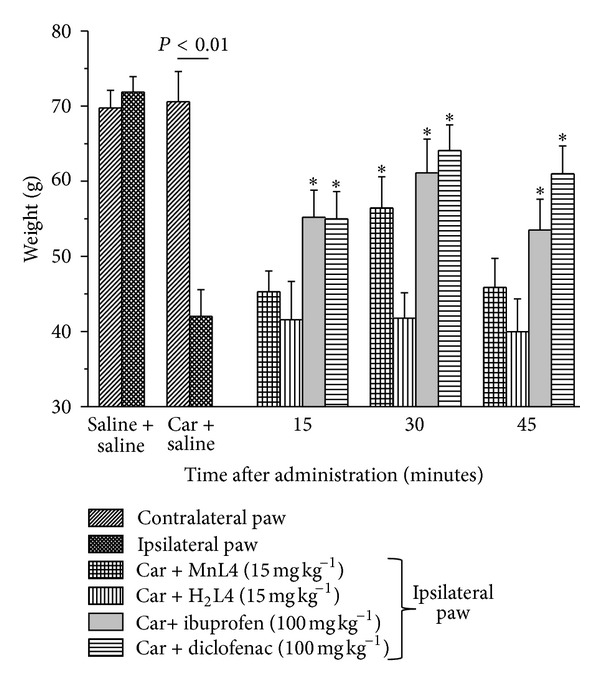
Effect of MnL4, H_2_L4, ibuprofen, and diclofenac on carrageenan-induced acute inflammatory pain. Carrageenan (Car, 1%, 100 *μ*L) was injected in the left posterior sole 3 hours before pain evaluation by the paw pressure test. Molecules or saline was administered i.p. at time 0 and measures were performed at time 15, 30, and 45 min. Values are the mean ± s.e.m. of 6 animals. ∗*P* < 0.05 versus the ipsilateral paw of carrageenan + saline group.

Three hours after the administration of carrageenan, all inflammatory signs were observed (paw swelling hyperaemia and hyperalgesia). The paw pressure test was used to measure pain. In ipsilateral paw (carrageenan + saline), the mechanical withdrawal threshold was significantly decreased as compared to the controlateral paw and control animals (saline + saline, Figure 1) and remained to the same value for at least 1 h. MnL4 (15 mg kg^−1^) significantly increased mechanical withdrawal threshold in the ipsilateral (carrageenan + MnL4) paw 30 min after its i.p. administration (Figure 1), but not modify the contralateral one (data not shown). Neither H_2_L4 (Figure 1), nor ibuprofen or diclofenac (not shown) at the same dosage of MnL4 were active, whereas ibuprofen and diclofenac at 100 mg kg^−1^ i.p. were effective (Figure 1).

### 3.2. Effects of MnL4 on CFA-Induced Inflammatory Arthritis

**Figure 2:**
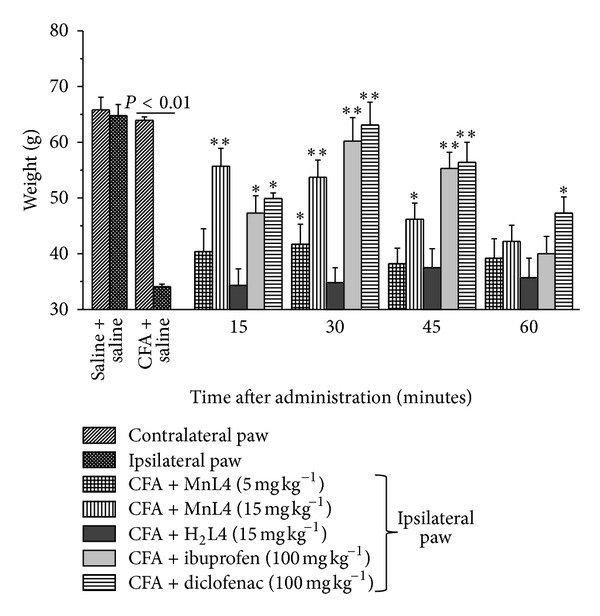
Effect of MnL4, H_2_L4, ibuprofen, and diclofenac on complete Freund's adjuvant-induced inflammatory arthritis. Complete Freund's adjuvant (CFA, 50 *μ*L) was injected in the left posterior tibiotarsal articulation 7 days before the pain evaluation by the paw pressure test. Molecules or saline was administered i.p. at the indicated doses at time 0 and measures were performed at time 15, 30, 45, and 60 min. Values are the mean ± s.e.m. of 6 animals. ∗*P* < 0.05 and ∗∗*P* < 0.01 versus the ipsilateral paw of CFA + saline group.

With the aim of testing the pharmacological activity of MnL4 in articular inflammatory damage resembling human rheumatoid arthritis [27], the SOD mimetic compound was evaluated in the CFA-model. The pain threshold was measured 7 days after intra-articular CFA injection by paw pressure and incapacitance tests. The mechanical withdrawal threshold in ipsilateral- (CFA + saline) treated paw was significantly reduced as compared to the controlateral paw and control animals (saline + saline). MnL4 (15 mg kg^−1^), 15 minutes after i.p. administration, increased the withdrawal threshold and was still effective after 45 minutes while at the dose of 5 mg kg^−1^ was effective 30 min after administration (Figure 2). Ibuprofen and diclofenac at 100 mg kg^−1^ i.p. were also active (Figure 2). H_2_L4 (Figure 2), ibuprofen, or diclofenac (not shown) was ineffective at 15 mg kg^−1^. Moreover, MnL4 significantly reduced hind paw unbalance in a time-dependent manner, being particularly effective 30 min after i.p. injection (Table 1).

### 3.3. Effect of MnL4 on MIA-Induced Osteoarthritis

**Figure 3:**
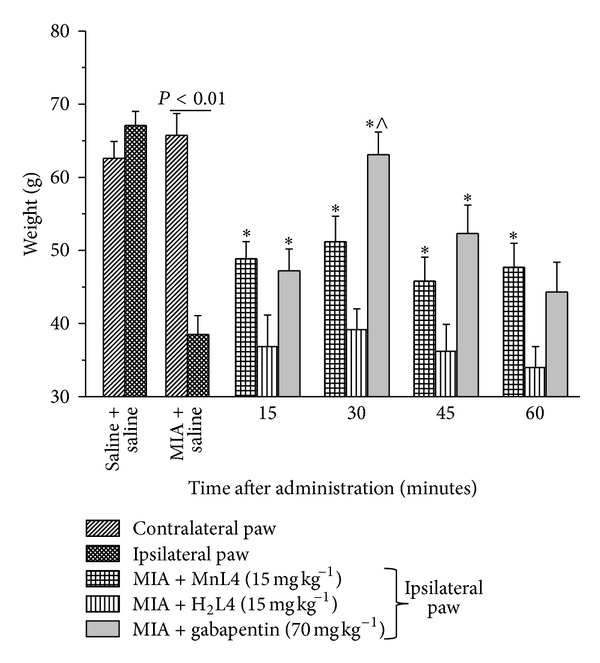
Effect of MnL4, H_2_ L4, and gabapentin (acutely administered) on monoiodoacetate- (MIA-) induced unilateral osteoarthritis. MIA (2 mg/25 *μ*L) was injected in the left posterior tibiotarsal articulation 14 days before pain evaluation by the paw pressure test. Molecules or saline was administered i.p. at time 0 and measures were performed at time 15, 30, 45, and 60 min. Values are the mean ± s.e.m. of 6 animals. ∗*P* < 0.05 versus the ipsilateral paw of MIA + saline group, ^∧^ *P* < 0.05 versus MIA + MnL4 at the same time.

The effectiveness of MnL4 was evaluated in the rat unilateral osteoarthritis induced by MIA according to two different protocols: acute i.p. administration (15 mg kg^−1^, 15–60 minutes before the test) or continuous subcutaneous infusion by osmotic minipumps (15 mg kg^−1^ day^−1^ for 14 days). Fourteen days after MIA, the weight tolerated on the ipsilateral paw (MIA + saline) was significantly reduced as compared to the controlateral paw and control animals (saline + saline, Figure 3). MnL4 (15 mg kg^−1^), 15 minutes after i.p. administration, increased the withdrawal threshold and was still effective after 60 minutes. At the same dosage, H_2_L4 was ineffective (Figure 3). Gabapentin (70 mg kg^−1^) showed a higher effectiveness than MnL4 30 min after administration, but was similarly active at the other times (Figure 3).

Moreover, MnL4 significantly reduced hind limb weight bearing alterations, being particularly effective 30 min after i.p. injection (Table 1).

**Figure 4:**
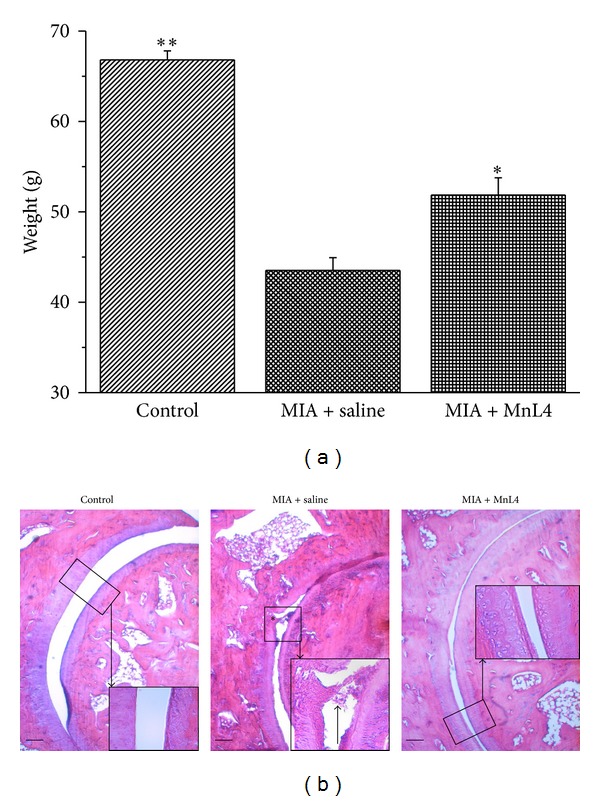
Effect of MnL4 (continuously infused for 15 days) on monoiodoacetate- (MIA-) induced unilateral osteoarthritis. MIA (2 mg/25 *μ*L) was injected in the left posterior tibiotarsal articulation 14 days before the pain evaluation by the paw pressure test. At day 0, a minipump containing MnL4 solution (15 mg kg^−1^ day) was implanted on the back of MnL4-treated rats. Values are the mean ± s.e.m. of 5 animals. Panel (a): pain behavior; ∗*P* < 0.05 and ∗∗*P* < 0.01 versus MIA + saline. Panel (b): effect of MnL4 (continuously infused for 15 days) on tibiotarsal articulation histopathology on MIA-induced osteoarthritis. Hematoxilin and eosin staining of longitudinal section of tibiotarsal joint. Pictures are representative of histological preparations from 5 animals per group. Bars = 100 *μ*m.

**Figure 5:**
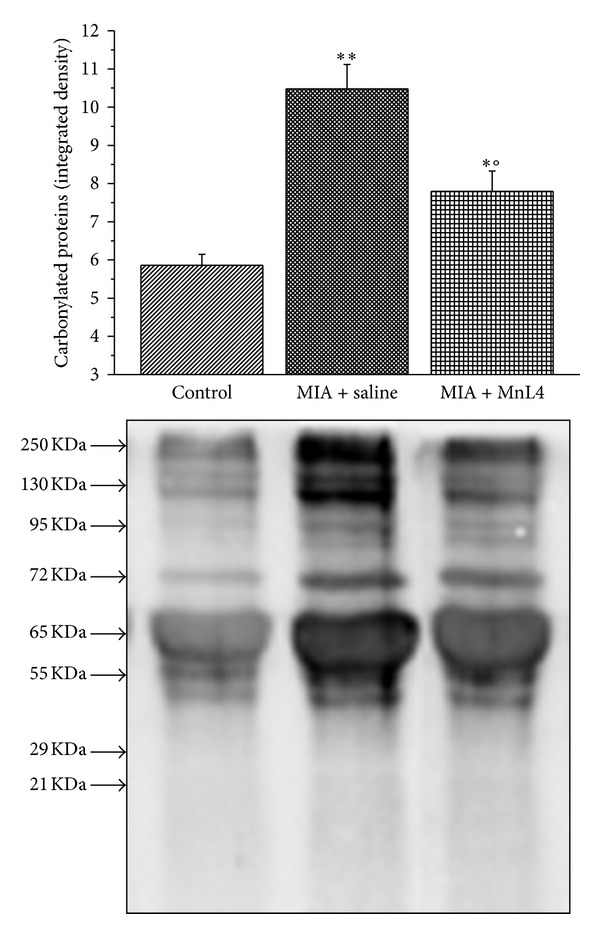
Effect of MnL4 (15 mg kg^−1^ continuously infused for 15 days) on plasma oxidation levels 14 days after MIA injection (2 mg/25 *μ*L in the left posterior tibiotarsal articulation on day 0). Immunoblot analysis was performed after a reaction with dinitrophenylhydrazine. Densitometric analysis and representative Western blot are shown. Ponceau-stained membranes were used as loading control. Each value represents the mean of 4 biological samples. ∗*P* < 0.05 and ∗∗*P* < 0.01 versus control rats; °*P* < 0.05 versus MIA + saline treatment.

MnL4 (15 mg kg^−1^ day^−1^) was also effective when continuously administered by s.c. route for 14 days (Figure 4(a)). This functional effect was accompanied by a substantial improvement of joint histopathology.  Figure 4(b) shows representative pictures of hematoxylin-eosin-stained longitudinal sections of tibiotarsal joints in the different experimental conditions: 14 days after injection, MIA caused intra-articular fibrin accumulation and extensive degeneration of the articular cartilage, that is, overall thinning, ulceration, and scarring. These changes resulted in a marked reduction of the intra-articular space compared to the normal joint (contralateral, control). Continuous s.c. administration of MnL4 prevented the appearance of these cartilage abnormalities and improved the intra-articular space. This beneficial effect of long-term MnL4 treatment was confirmed by the dosage of TNF-*α* plasma levels. As reported in Table 2, TNF-*α* was significantly increased in MIA + saline-treated rats at day 14 compared to naïve animals; MnL4 completely prevented MIA-induced TNF-*α* elevation. Moreover, in MIA + saline-treated rats, systemic oxidative damage was also present, as evaluated by the carbonylation of plasma proteins. In fact, on the 14th day, plasma-carbonylated proteins increased up to twice the basal level of naive animals (5.9 ± 0.29; densitometric arbitrary units). MnL4 (15 mg kg^−1^ day^−1^) significantly reduced this oxidation parameter (Figure 5).

### 3.4. Effect of MnL4 on Lipid Peroxidation in Human-Cultured Chondrocytes

**Figure 6:**
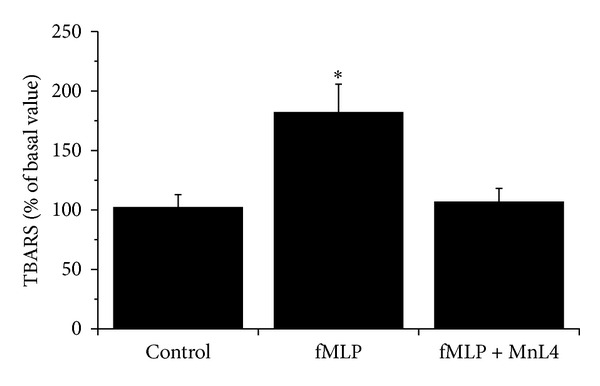
Effect of 10^−7 ^M MnL4 on the lipid peroxidation (TBARS) induced by superoxide anion in human chondrocytes. Human chondrocytes were coincubated with RAW 264.7 with or without 10^−7^ M MnL4. RAW 264.7 were activated by 10^−7^ M fMLP for 4 h to produce superoxide anions. Values are expressed as percentage of control values (human chondrocytes coincubated with unstimulated RAW 264.7). TBARS in control value was 1.65 ± 0.18 *μ* moles mg^−1^ of total proteins. Each value represents the mean of 5 experiments, performed using chondrocytes isolated from 3 patients. ∗*P* < 0.05 versus all other conditions.

In order to study the effect of MnL4 on an ROS attack in HCs, we performed experiments in a coculture system of HCs and mouse leukaemic monocyte macrophage cells (RAW 264.7). This experimental set allowed us to study the effect of RAW 264.7-produced O_2_^•−^ on HCs lipid peroxidation. In basal conditions (without coculture with RAW 264.7), membrane lipid peroxidation of HCs (expressed as TBARS) was 1.24 ± 0.14 *μ*mol  mg^−1^ of proteins. This value was not significantly modified when HCs were incubated with unstimulated RAW 264.7 (1.65 ± 0.18 *μ*mol  mg^−1^ of proteins, control) but was markedly and significantly increased up to 3.0 ± 0.54 *μ*mol  mg^−1^ of proteins when RAW 264.7 were stimulated with 10^−7^ M fMLP (Figure 6). When cells were preincubated with 10^−7^ M MnL4, lipid peroxidation was totally prevented.

## 4. Discussion

Inflammatory conditions (and in particular, joint diseases) induce an increase in ROS which have a deleterious role in erosion, osteoarticular degeneration, and pain. Conversely, ROS increase inflammatory mediators [28]. ROS are also implicated in persistent pain behavior as already demonstrated by several authors [25, 29, 30]. Therefore, molecules able to reduce O_2_^•−^ can be used to reduce pain and inflammation.

Following this line of reasoning, extractive or recombinant SOD seems to be the most valid choice for such a targeted therapeutic approach [31]. However, its clinical use is hampered by multiple factors, including instability, limited cellular accessibility, immunogenicity, short half-life, and high production costs [32, 33]. Because of these limitations, SOD mimetic compounds have been proposed as appropriate strategies in many degenerative pathological conditions [32], and pharmacological research has highlighted low molecular weight compounds, such as the antioxidant Tempol [34] and the Mn^II^ chelates with organic scaffolds [17, 35], capable of catalyzing O_2_^•−^ decomposition like authentic SOD. Tempol and Mn^II^ complexes with pentaazamacrocycles, salen-, and porphyrin-based scaffolds have been reported to reduce inflammation and pain in different animal models of articular diseases [9, 34, 36, 37]. The SOD mimetic compound MnL4 has already been characterized as a membrane-permeable, highly effective scavenger compound [15], possessing anti-inflammatory properties in a model of allergic asthma [16]. Therefore, we studied it using a panel of *in vivo* rat models of articular pain induced by acute and chronic inflammation. 

Intra-articular injection of MIA provides a rodent model of monolateral osteoarthritis with features resembling those seen clinically. These include synovial thickening, loss of cartilage, formation of osteophytes, and eventual fibrillation of cartilage [18, 38, 39]. Morphological alterations are associated with a persistent inflammatory pain which, starting from the 14th day after MIA injection, possesses a neuropathic component [20]. Nonsteroidal anti-inflammatory drugs such as diclofenac can reduce MIA-dependent pain during the first inflammatory phase, but they are ineffective in the degenerative neuropathic phase [40], while gabapentin, an antiepileptic molecule widely used to treat neuropathic pain in adult patients [41], is effective [20].

In the MIA model, acutely administered MnL4 (15 mg kg^−1^) causes a prolonged (60 min) reduction of pain sensitivity during the phase when the neuropathic component prevails over the inflammatory one and its efficacy is quite similar to that of gabapentin administered at typical dosage [20]. Its parent compound, H_2_L4, which lacks ROS-scavenging effects, is totally ineffective. 

Although we injected MIA in tibiotarsal articulation, several characteristics of our model resemble those observed after MIA knee injection. Indeed, after 14 days from MIA injection, the neuropathic component of pain predominates as demonstrated by the high effectiveness of gabapentin. Moreover, the histological analysis confirms a degeneration pattern of the tibiotarsal joint similar to that described for knee [23, 42]. The performed model permits us to directly compare MnL4 as pain reliever in MIA and CFA. 

In the same osteoarthritis model, MnL4, continuously infused by an osmotic pump (chronic administration), increases the pain threshold and ameliorates tibiotarsal joint histopathological parameters. Moreover, in blood samples obtained at the same stage of joint degenerative changes, the SOD mimetic compound prevents the significant, TNF-*α* increase induced by MIA and reduces protein carbonylation. The proinflammatory cytokine, TNF-*α*, is a critical mediator in osteoarthritis and rheumatic disease. Its serum level is linearly related to disease activity clinical score in patients with rheumatoid arthritis, and it has been proposed as clinical marker of this pathology [43]. TNF-*α* upregulation is a consequence of NF*κ*B nuclear translocation which can be due to the ROS-activated intracellular signaling cascade [44]. Carbonylation of proteins is an irreversible oxidative damage. Carbonyl groups are introduced into protein side chains by a site-specific mechanism often leading to a loss of protein function. It is considered a widespread indicator of severe oxidative damage and disease-derived protein dysfunction [45]; its increase has been described in the plasma of human subject affected by systemic rheumatic diseases [46]. 

CFA-induced inflammatory arthritis in rats presents similar features to rheumatoid arthritis [27]. CFA-induced inflammatory arthritis starts between the 3rd and 7th days after inoculation; at this time, the pain threshold is significantly decreased [47], while sensory neuron firing is increased [48] leading to changes in gene expression and sensitization of the nervous system. These functional alterations contribute to the pain associated with joint injuries [49]. Seven days after CFA injection, 15 mg kg^−1^ MnL4, acutely administered before the behavioral tests, increases the pain threshold for at least 45 min and prevents the hind limb weight bearing alterations whereas the scaffold congener of MnL4 is totally ineffective. 

Since acute inflammation occurs at the initial stage of articular diseases, we tested MnL4 in carrageenan-induced paw acute edema. In this condition, 30 min after administration, MnL4 enhances the pain threshold, decreasing mechanical hypersensitivity by about 50%. However, the effectiveness of MnL4 in this model is short lasting. At the same dosage (15 mg kg^−1^), the well-known anti-inflammatory NSAIDs ibuprofen and diclofenac are ineffective, being their anti-inflammatory activity observed at higher dosages (100 mg kg^−1^) currently used in animal tests [18, 19]. 

Many of the effects of MnL4 are in agreement with the antioxidant property of the compound [15, 16]: accumulating evidence indicates that the production of ROS is increased in the nociceptive system during persistent inflammatory and neuropathic pain [50]. Since ROS have also been implicated in chondrocyte degeneration and death [4], we tested MnL4 activity against the oxidative stress induced by O_2_^•−^ in isolated human chondrocytes. According to previous data of our laboratory on reproducibility and effectiveness of RAW 264-7 in producing O_2_^•−^ after fMLP stimulation [15], we coincubated human chondrocytes with RAW 264-7. This experimental condition simulates an ROS attack on chondrocytes by infiltrating inflammatory cells. MnL4 at 10^−7^ M can totally prevent lipid peroxidation, suggesting an important contribution to joint protection. 

In conclusion, MnL4 behaves as a potent pain reliever compound both in arthritis models and, to a lesser extent, in acute inflammation. This effect is not related to a direct inhibition of cyclooxygenase enzymes as already described [15] but, conceivably, related to the SOD mimetic property of the molecule as also demonstrated on HCs. The mechanism by which MnL4 acts after chronic and acute administration may be somewhat different. Namely, chronically administered MnL4 may prevent tissue degenerative alterations induced by the oxidative stress and reduce a persistent inflammatory pain via a direct antioxidant mechanism; while in acute administration, it may decrease the nociceptive nervous fiber activation induced by the local production of ROS [28, 50]. Given these properties and the low toxicity of the molecule, MnL4 is a novel compound potentially suited for the treatment of inflammatory and neuropathic pain.

## Supplementary Material

Figure S1: Chemical structures of MnL4 and of the scaffold H_2_L4.

## Figures and Tables

**Table 1:** Effect of MnL4 on hind limb weight bearing alterations induced by CFA or MIA.

Treatment	Δ Weight (g) (contralateral minus ipsilateral paw)				
	0 min	15 min	30 min	45 min	60 min
Control (saline + saline)	5.2 ± 2.3	3.1 ± 1.9	3.8 ± 2.5	4.2 ± 3.0	6.3 ± 3.1
CFA + 15 mg kg^−1^ MnL4	58.8 ± 1.6^*∧∧*^	37.9 ± 2.1*	28.7 ± 1.9**	34.8 ± 2.0*	40.6 ± 2.3*

Control (saline + saline)	3.2 ± 1.2	−2.3 ± 2.1	1.5 ± 3.1	−3.0 ± 2.8	3.8 ± 2.5
MIA + 15 mg kg^−1^ MnL4	61.3 ± 2.3^*∧∧*^	30.2 ± 3.1*	20.1 ± 3.6**	40.3 ± 1.1*	55.3 ± 2.9

Hind limb weight bearing alterations were evaluated in rats by incapacitance test. In the absence of hind limb injury, rats applied an equal weight on both hind limbs, whereas an unequal distribution of the weight on hind limbs indicated a monolateral decreased pain threshold. CFA was injected 7 days before the test, MnL4 was acutely i.p. administered at time 0 min; MIA was injected 14 days before the test, MnL4 was acutely i.p. administered at time 0 min.

^*∧∧*^ *P*  <  0.01 in respect to control (saline + saline) group; **P  <  0.01 and *P  <  0.05 with respect to the 0 min value of the same treatment.

**Table 2:** TNF*α* plasma levels in control, MIA- and MnL4 + MIA-treated rats.

	Control (saline + saline)	Saline + MIA	MnL4 + MIA
pg/mL	5.37 ± 1.77	16.86 ± 2.02*	4.75 ± 1.69

Monoiodoacetate (MIA, 2 mg/25 *µ*L) or saline was injected in the left posterior tibiotarsal articulation 14 days before the test. At day 0, a minipump containing MnL4 solution (15 mg kg^−1^ day) was implanted on the back of MnL4 + MIA-treated rats. TNF*α* levels were measured in plasma samples by ELISA.

*P  <  0.01 versus saline + saline and the MnL4 + MIA groups.
