# Supplementary figures and images for: Therapeutic Effects of the Superoxide Dismutase Mimetic Compound MnIIMe2DO2A on Experimental Articular Pain in Rats
Source: Mediators Inflamm. 2013 Sep 1;2013:905360. doi: 10.1155/2013/905360 (PMC3690261; doi:10.1155/2013/905360)

## Slide 1
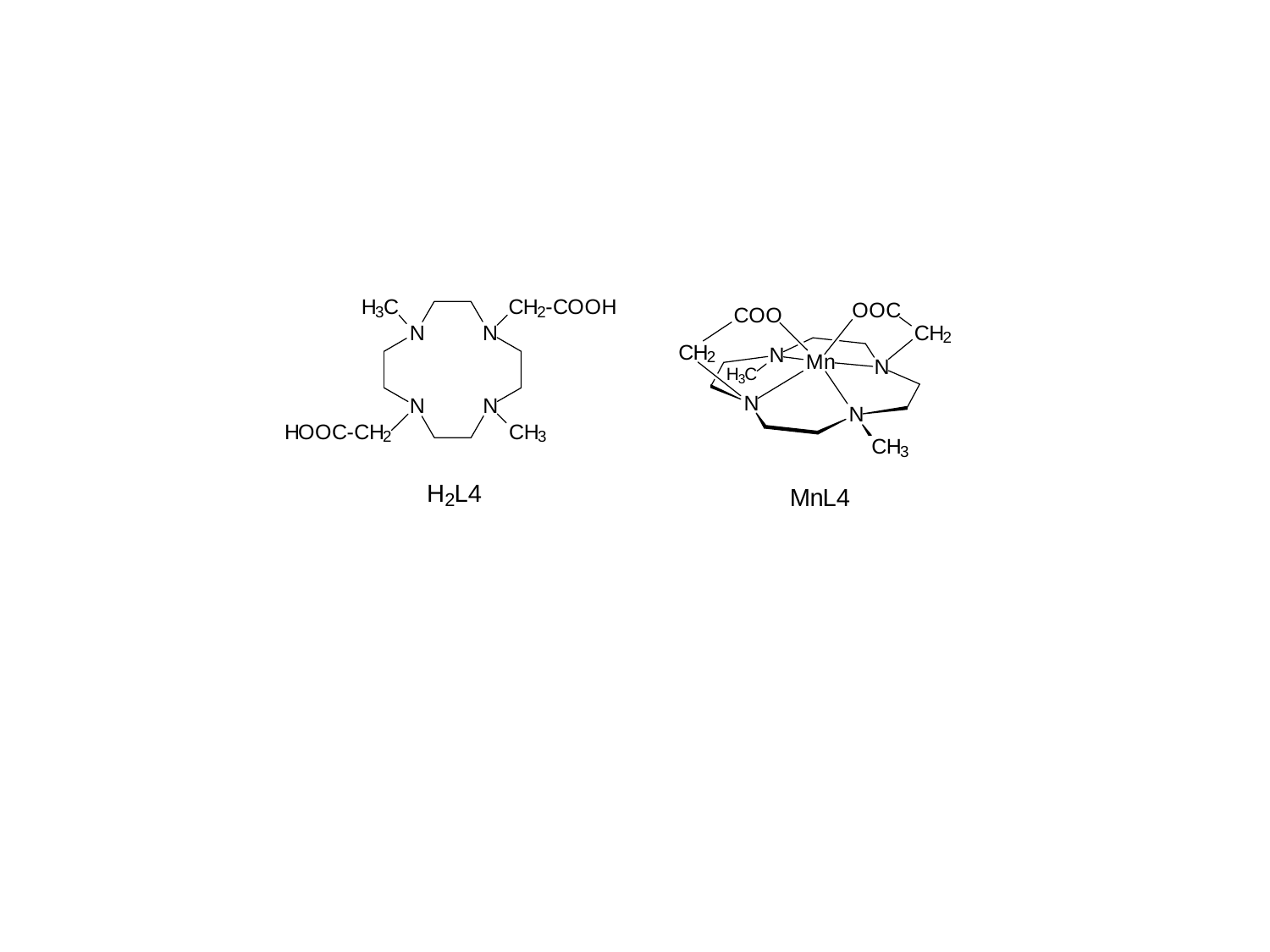

Supplement: Supplementary file 1 — Figure S1: Chemical structures of MnL4 and of the scaffold H2L4. [file 905360.f1.ppt]
